# Methyl-β-cyclodextrin suppresses the monocyte-endothelial adhesion triggered by lipopolysaccharide (LPS) or oxidized low-density lipoprotein (oxLDL)

**DOI:** 10.1080/13880209.2021.1953540

**Published:** 2021-08-06

**Authors:** Guo Chen, Yun Zhou, Wendiao Zhang, Ying Qin, Bo Wei, Yanan Sun, Yong Chen

**Affiliations:** a College of Life Sciences, Nanchang University, Nanchang, Jiangxi, China; b Jiangxi Key Laboratory for Microscale Interdisciplinary Study, Institute for Advanced Study, Nanchang University, Nanchang, Jiangxi, China

**Keywords:** Cyclodextrins (CDs), human umbilical vein endothelial cells (HUVECs), THP-1, atherosclerosis, tumour, NF-κB, cell adhesion, lipid raft

## Abstract

**Context:**

Recent studies demonstrated the anti-atherosclerotic efficacy of cyclodextrin. However, it remains unclear whether cyclodextrin exerts the anti-atherosclerotic effect via regulating monocyte-endothelial adhesion.

**Objective:**

To answer that question by recruiting methyl-β-cyclodextrin (MβCD) as a cyclodextrin representative.

**Materials and methods:**

Human umbilical vein endothelial cells (HUVECs) were not treated, or treated with 1 µg/mL liposaccharide (LPS) or 50 µg/mL oxidized low-density lipoprotein (oxLDL) for 12 h, 5 mM MβCD for 1 h, and LPS/oxLDL (1 and 50 µg/mL, respectively for 12 h) plus MβCD (5 mM for 1 h), respectively. The effects of MβCD on LPS/oxLDL-triggered monocyte-endothelial adhesion and related molecules in signalling pathways were evaluated via confocal microscopy, flow cytometry, RT-PCR, western blotting, and cell adhesion assay.

**Results:**

MβCD with an IC_50_ of 27.66 mM (1 h treatment) exerted no significant cytotoxicity at ≤5 mM for ≤2 h. Compared with the control, both LPS and oxLDL induced an ∼2–3-fold increase in adhesion molecule expression (ICAM-1 and VCAM-1 at protein and mRNA levels) and NF-κB phosphorylation (p-NF-κB/pP65), an increase in IκB kinase (IKK), and a decrease in phosphorylated protein kinase B (p-Akt), respectively. Moreover, more monocytes (2-fold higher for LPS and 15% higher for oxLDL) were attached on LPS/oxLDL-stimulated HUVECs. 5 mM MβCD reversed the LPS/oxLDL-induced changes back to the control levels.

**Conclusions:**

MβCD significantly suppresses the LPS/oxLDL-triggered monocyte-endothelial adhesion by downregulating adhesion molecule expression probably via LPS-IKK-NF-κB or oxLDL-Akt-NF-κB pathway. This study demonstrates a potential mechanism of the anti-atherosclerotic efficacy of cyclodextrin from the angle of monocyte-endothelial adhesion.

## Introduction

Cyclodextrins (CDs) are cyclic oligosaccharides produced by the enzymatic hydrolysis of starch. Cyclodextrins are made up of various numbers of α-(1,4)-linked glucopyranose subunits, such as α-cyclodextrin (α-CD; six subunits), β-cyclodextrin (β-CD; seven subunits), γ-cyclodextrin (γ-CD; eight subunits), among others (Duchene and Bochot 2016). Due to the barrel-like structure with a hydrophobic internal cavity surrounded by a hydrophilic outer surface, cyclodextrins have long been developed as well-known, highly-efficient food/drug carriers/excipients for improving the water solubility of foods/drugs (Li et al. 2007; 2014; Saokham et al. 2018; Carneiro et al. 2019). To improve the water solubility, various cyclodextrin derivatives have been developed, such as methyl-β-cyclodextrin (MβCD), a widely studied derivative of β-CD particularly in cell experiments (Ashrafzadeh and Parmryd 2015; Zhang et al. 2019).

Atherosclerosis is a chronic cardiovascular disease characterized by the formation of atherosclerotic plaques and the narrowing of the arterial lumen. In recent years, accumulating evidence supports that cyclodextrins *per se* have anti-atherosclerotic efficacy (Zimmer et al. 2016; Ao et al. 2016a; Ao and Chen 2017; Sakurai et al. 2017; Pilely et al. 2019; Wang et al. 2019). Different mechanisms have been reported, such as enhanced cholesterol efflux from atherosclerotic plaques/macrophages (Zimmer et al. 2016), inhibited oxidation of plasma LDL (Ao et al. 2016a; Ao and Chen 2017), increased level of plasma HDL (Wang et al. 2019), reduced cholesterol crystal-induced complement activation (Pilely et al. 2019), and modified gut flora (for oral administration) (Sakurai et al. 2017).

During the initiation or development of atherosclerosis, monocytes in the blood circulation adhere onto the endothelial cells, infiltrate into the sub-endothelial space (i.e., the arterial tunica intima), and differentiate to macrophages. Therefore, the monocyte-endothelial adhesion is an important initial step of atherogenesis. In one of our previous studies, we found that MβCD could impair the monocyte-adhering ability of normal endothelial cells by influencing adhesion molecules and cytoskeleton (Ao et al. 2016b). However, it is unclear whether cyclodextrins exert the anti-atherosclerotic efficacy *via* influencing the monocyte-endothelial adhesion during atherogenesis at which situation endothelial cells are generally activated by some stimuli, such as lipopolysaccharide (LPS) or oxidized low-density lipoprotein (oxLDL). In this study, we sought to test this possibility by using endothelial cells stimulated with LPS or oxLDL as an *in vitro* cell model of atherosclerosis.

## Materials and methods

### Cell culture

Human umbilical vein endothelial cells (HUVECs), purchased from Procell Life Science & Technology Co., Ltd. (Wuhan, China), were cultured in Ham’s F-12K medium (Procell Life Science & Technology Co., Ltd.) supplemented with 10% (w/v) foetal bovine serum (FBS), 0.1 mg/mL heparin, 0.03-0.05 mg/mL endothelial cell growth factors (EGFs), 100 U/mL penicillin, and 100 µg/mL streptomycin. Human THP-1 monocytic leukaemia cells purchased from KeyGen Biotech Co., Ltd. (Nanjing, China) were cultured in Dulbecco’s modified Eagle’s medium (DMEM; Sigma, USA) supplemented with 10% FBS, 100 U/mL penicillin, and 100 µg/mL streptomycin. Approximately the fifth passage of cells was utilized for all experiments.

### Cell treatments

For the experiments in which the only activation was performed, HUVEC cells were incubated with 0, 0.1, 0.5, 1, and 10 µg/mL lipopolysaccharide (LPS; Sigma), respectively for 12 h at 37 °C or with 0, 5, 25, 50, and 100 µg/mL oxidized low-density lipoprotein (oxLDL; Yiyuan Biotechnologies, Guangzhou, China), respectively for 12 h at 37 °C. For the experiments in which both activation and MβCD treatment were performed, HUVECs were first activated with 1 µg/mL LPS or 50 µg/mL oxLDL for 12 h at 37 °C, rinsed twice with phosphate-buffered serum (PBS) and then treated with 5 mM MβCD (Sigma, USA) for 0.5–1 h at 37 °C. After washing twice with PBS, the samples were subjected to the following experiments.

### Detection of ICAM-1/VCAM-1 expression by confocal microscopy

For the staining of intercellular cell adhesion molecule-1 (ICAM-1) or vascular cell adhesion molecule-1 (VCAM-1) on cell surfaces, the treated and washed cells were fixed with 4% paraformaldehyde for 30 min at room temperature, rinsed three times with PBS, blocked with 2% bovine serum albumin (BSA) for 1 h, incubated with anti-ICAM-1 mAb or anti-VCAM-1 mAb (eBioscience, USA) for 1 h, rinsed three times with PBS, and incubated with AlexaFluor488-conjugated goat anti-mouse IgG (Life Technologies, USA) for 1 h. After washing three times with PBS, the samples were subjected to confocal microscopy.

An LSM710 confocal microscope (Carl Zeiss, Oberkochen, Germany) equipped with a Zeiss inverted microscope and a Zeiss 40× (0.75 NA) Plan-Neofluar objective lens were used for fluorescence imaging.

### Detection of ICAM-1 expression by flow cytometry

For the staining of ICAM-1 in cells, the treated and washed cells were blocked with 2% BSA for 1 h at 37 °C, incubated with anti-ICAM-1 mAb for 1 h at 37 °C, washed three times with PBS, and incubated with AlexaFluor488-conjugated goat anti-mouse IgG for 1 h at 37 °C. After washing three times with PBS, the cells were digested with trypsin (Sigma), harvested via centrifugation at 1,000 rpm for 3 min, and fixed with 4% paraformaldehyde for 30 min. After washing with PBS via centrifugation, the cells were subjected to flow cytometry. A FACSCalibur flow cytometer (BD Biosciences, USA) was utilized for flow cytometric acquisition and analysis.

### Measurement of ICAM-1 mRNA expression

HUVECs were activated with or without 1 µg/mL LPS or 50 µg/mL oxLDL for 12 h at 37 °C, rinsed twice with PBS, treated with 5 mM MβCD for 0.5–1 h, rinsed again with PBS, and subjected to the detection of ICAM-1 mRNA expression. The extraction of total RNA via the E.Z.N.Z. Total RNA Kit (OMEGA, USA), the synthesis and amplification of cDNA via the HiFiScript cDNA Synthesis Kit (ComWin Biotech Co., Ltd., Beijing, China), and the used primer sequences for ICAM-1 and GAPDH (an internal reference) have been reported in our previous studies (Ao et al. 2019; Zhang et al. 2019). The cDNA was detected by electrophoresis on a 0.8% agarose gel at 110 V for 25 min and imaged using a gel imaging system (GelDoc 2000, Bio-Rad, CA).

### MTT assay

MTT assay was performed as previously reported (Zeng et al. 2013). Briefly, HUVECs in each well of a 96-well plate were treated with different concentrations (0-100 mM as indicated) of MβCD (Sigma, USA) for 1 h at 37 °C or at a specific concentration (5 mM) for different periods of time (0, 1, 2, 4, and 8 h, respectively). After washing with PBS, fresh medium (100 µL) and MTT (5 mg/mL, 20 µL) were added into each well and incubated with the cells for 4 h. After reaction termination, the solution was removed from each well, and diethyl sulfoxide (DMSO, 100 µL) was added and incubated with the cells at 37 °C for 10 min. A microplate reader (Rayto, China) was utilized to measure the optical density (OD) at 490 nm.

### Western blot

The treated and washed cells were harvested via centrifugation at 1,000 rpm for 3 min. Protein extraction was performed according to the manufacturer’s introduction by using Nc-nuclear/cytoplasmic protein extraction Kit (ComWin Biotech Co., Ltd., Beijing, China). The extracted proteins were denatured by heating at 95 °C. The total protein concentration was quantified by a BCA protein assay. Protein samples in loading buffer were separated, transferred to nitrocellulose (NC) membranes, immune-stained, and chemiluminescent detected as reported in our previous study (Ao et al. 2019). The monoclonal antibodies against the key molecules of LPS and oxLDL pathways including P65, phosphorylated P65 (pP65), IKKα, AKT1, and phosphorylated AKT1 (pAKT1), as well as anti-GAPDH (an internal control) mAb and horseradish peroxidase (HRP)-conjugated goat anti-rabbit IgG, were all purchased from Abcam (UK).

### Monocyte-endothelial cell adhesion assay

Confluent HUVECs were activated with or without 1 µg/mL LPS or 50 µg/mL oxLDL for 12 h at 37 °C, rinsed twice with PBS, treated with 5 mM MβCD for 0.5-1 h, rinsed again with PBS, and cultured in medium. Monocyte-endothelial cell adhesion assay was performed as previously reported (Ao et al. 2016b). Briefly, THP-1 monocytic cells were stained with CellTracker Red CMTPX (Life Technologies, USA) for ∼30 min at 37 °C and washed twice with serum-free medium to remove excess dyes. Then the stained THP-1 cells were added onto the LPS-/oxLDL-activated, MβCD-treated HUVEC cell monolayer and co-cultured for 2 h at 37 °C in the CO_2_ incubator. After the removal of unattached THP-1 cells via washing with PBS, the cells were imaged under the confocal microscope (excitation and emission wavelengths for CellTracker Red CMTPX were 577 and 602 nm, respectively) and the amount of attached THP-1 cells on the endothelial cell monolayer in each field was quantified.

### Statistical analysis

All data from at least three independent experiments were expressed as the mean ± *SD*. Statistical analysis was performed via Student’s *t*-test or one-way ANOVA. A difference was considered as statistically significant when *p* < 0.05 (*p < 0.05; ***p < 0.001; ****p < 0.0001 compared with the corresponding group).

## Results

### Both LPS and oxLDL enhanced ICAM-1 expression in a concentration-dependent manner

**Figure 1.**
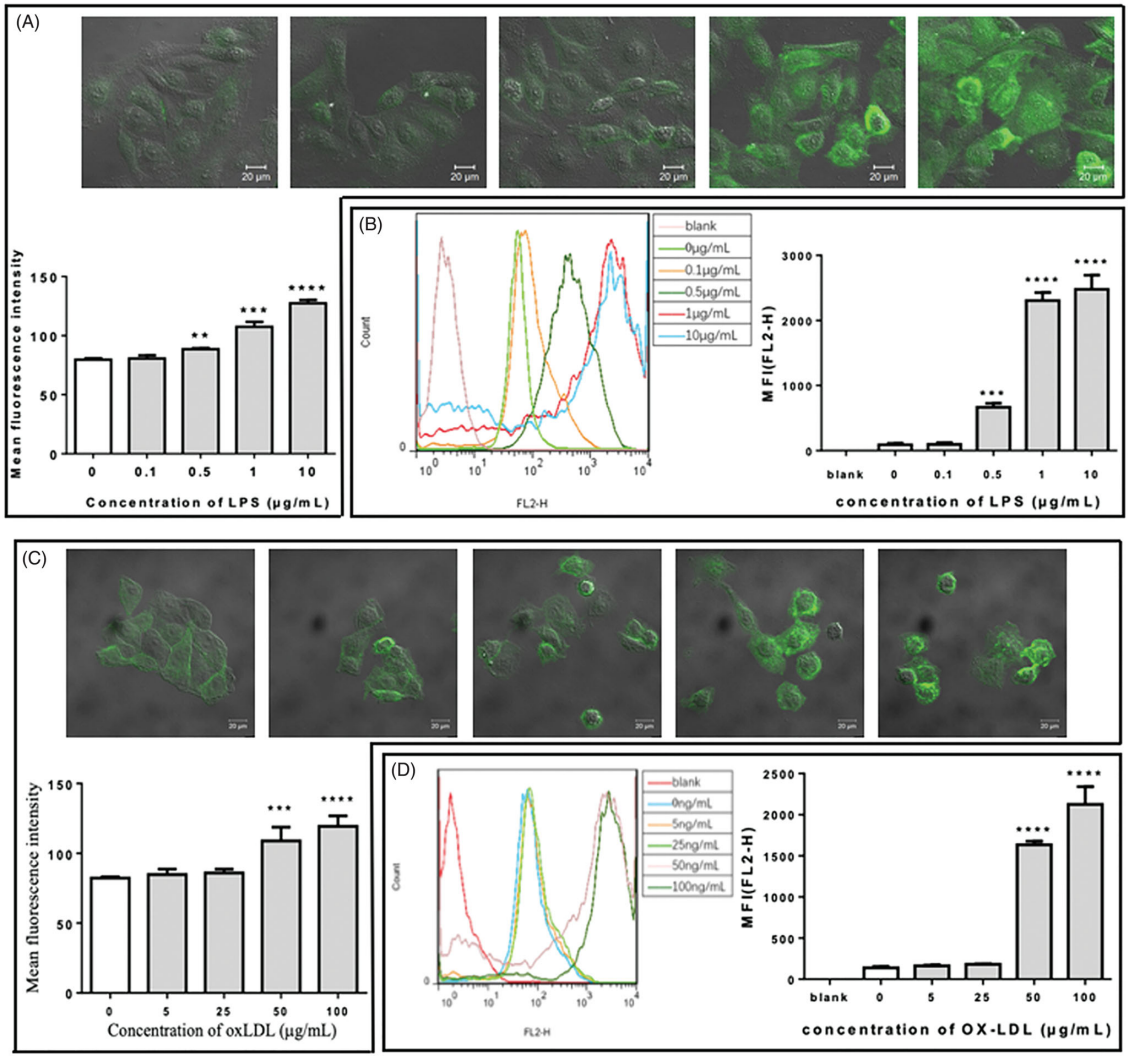
Effect of lipopolysaccharide (LPS) or oxidized low-density lipoprotein (oxLDL) at different concentrations on the expression of ICAM-1 in HUVECs. (A) Confocal microscopic data of HUVECs treated with LPS at different concentrations (0, 0.1, 0.5, 1, and 10 µg/mL, respectively). Upper panel: representative confocal images; lower panel: quantification of the mean fluorescence intensity (MFI). (B) Flow cytometric data of LPS-treated HUVECs. Left panel: representative flow cytometric data; right panel: quantification of the mean fluorescence. (C) Confocal microscopic data of HUVECs treated with oxLDL at different concentrations (0, 5, 25, 50, and 100 µg/mL, respectively). (D) Flow cytometric data of oxLDL-treated HUVECs. **p < 0.01; ***p < 0.001; ****p < 0.0001 compared with the control (*n* = 3).

Before the study on MβCD treatment, the effects of LPS and oxLDL on the expression of ICAM-1 in human umbilical vein endothelial cells (HUVECs) were confirmed by using confocal microscopy and flow cytometry (Figure 1). The data show that both LPS and oxLDL enhanced ICAM-1 expression and that ≥0.5 µg/mL LPS or ≥50 µg/mL oxLDL for 12 h exerted a statistically significant effect. Therefore, ∼1 µg/mL LPS or 50 µg/mL oxLDL for 12 h was applied to stimulate endothelial cells in the following experiments.

### MβCD reversed the LPS-/oxLDL-induced effect on ICAM-1/VCAM-1 expression

**Figure 2.**
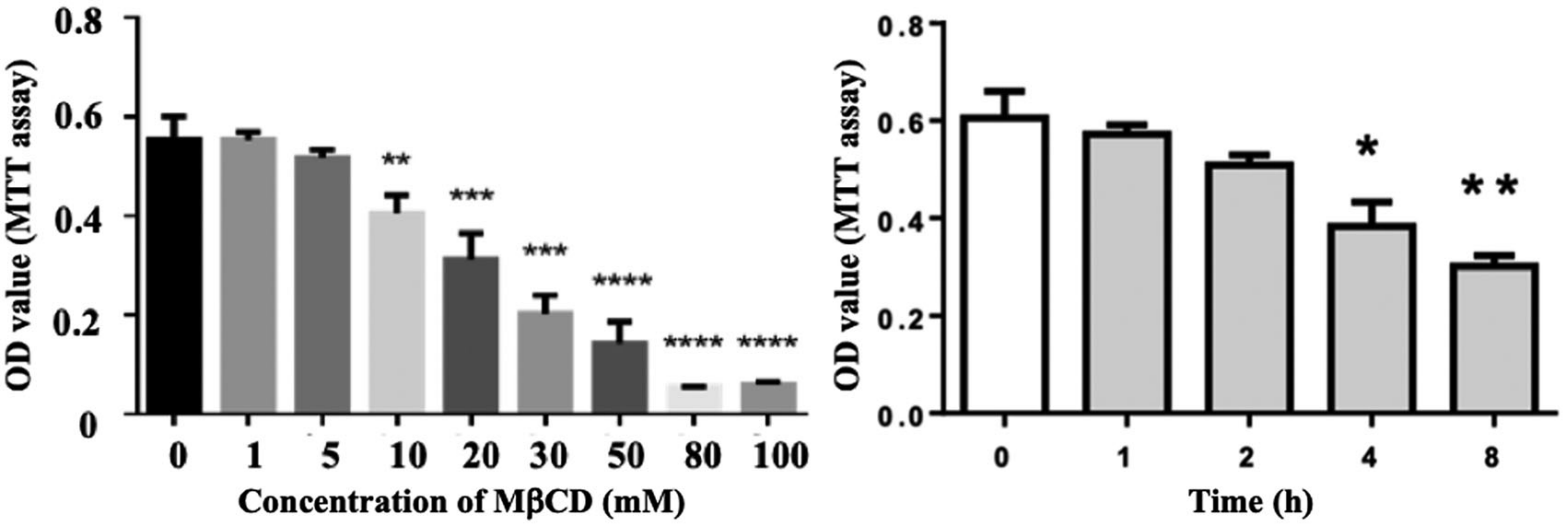
Effect of MβCD on the viability of HUVECs detected by MTT assay. Left: the cells were treated with MβCD at different concentrations (0–100 mM as indicated) for 1 h; right: the cells were treated with 5 mM MβCD for different periods of time (0, 1, 2, 4, and 8 h, respectively). *p < 0.05; **p < 0.01 compared with the control (left: *n* = 3; right: *n* = 4); ***p < 0.001; ****p < 0.0001.

Before the MβCD study on cell-to-cell adhesion, the effect of MβCD on cell viability of HUVECs was evaluated via MTT assay. Figure 2 shows that MβCD at a concentration of ≤5 mM for ≤2 h exerted no statistically significant cytotoxicity and that MβCD has an IC_50_ of 27.66 mM for 1 h treatment. In the following experiments, ∼5 mM MβCD for 1 h (a condition that is not cytotoxic) was applied to treat endothelial cells.

**Figure 3.**
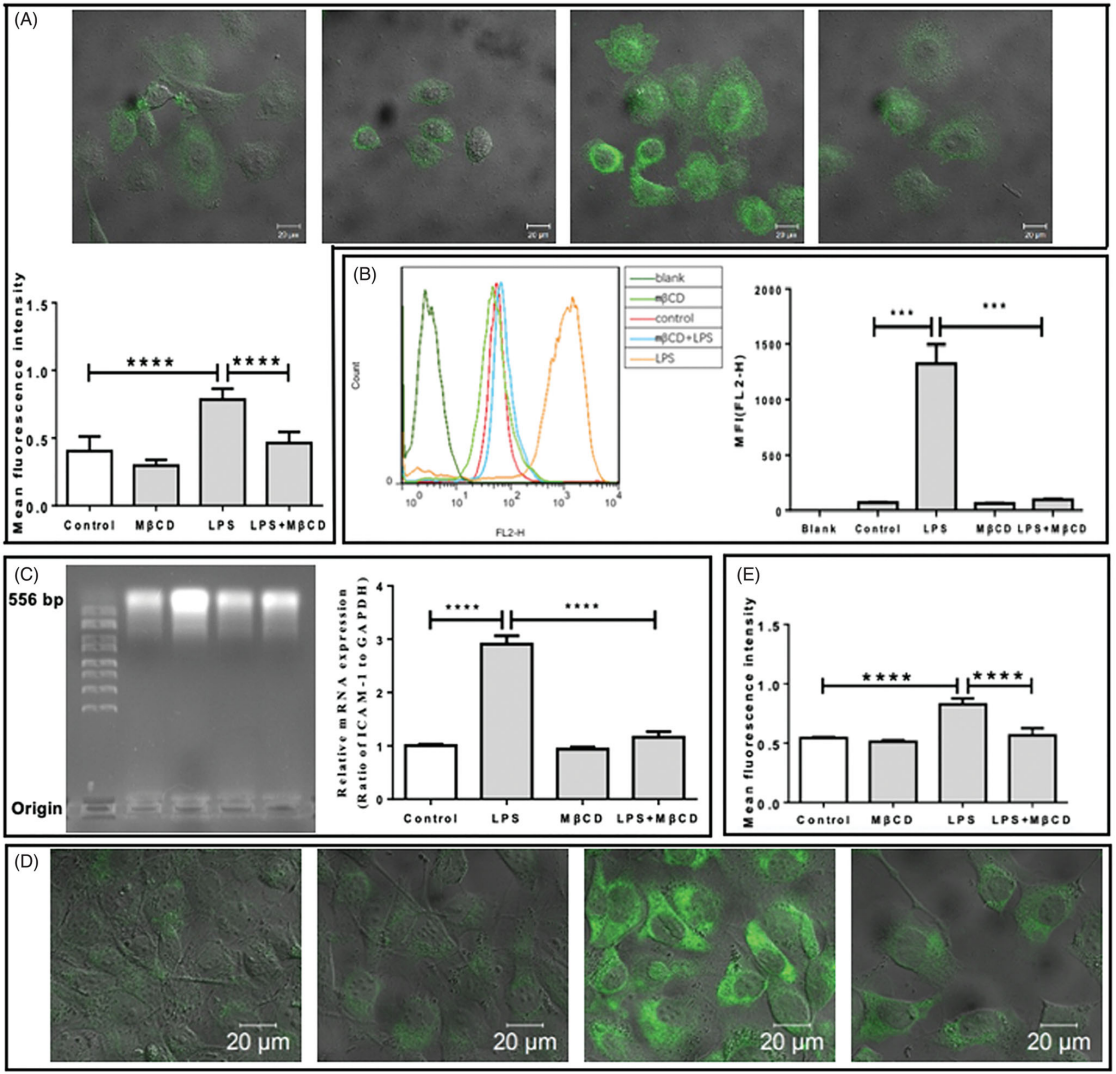
Inhibitory effect of MβCD on the LPS-induced expression of adhesion molecules in HUVECs. Four groups are categorized: HUVECs without treatment (control), treated with MβCD only (at 5 mM at 37 °C for 1 h), with LPS only (at 1 µg/mL at 37 °C for 12 h), and with both LPS and MβCD, respectively. (A) Confocal microscopic imaging of ICAM-1 molecules on cell surfaces (upper panel: representative confocal images; lower panel: quantification of MFI). (B) Flow cytometric detection of ICAM-1 molecules in cells (left panel: representative flow cytometric data; right panel: quantification of MFI). (C) The detection of ICAM-1 mRNA expression in cells. Left panel: agarose gel electrophoretic image; right panel: quantification of relative mRNA expression (ratio of ICAM-1 to GAPDH). (D) Representative confocal microscopic images of fluorescently stained VCAM-1 molecules on cell surfaces. From left to right: control, MβCD only, LPS only, and LPS + MβCD, respectively. (E) Quantification of VCAM-1 MFI. ***p < 0.001; ****p < 0.0001 compared with the corresponding group (*n* = 5 in A; *n* = 3 in B,C,E).

**Figure 4.**
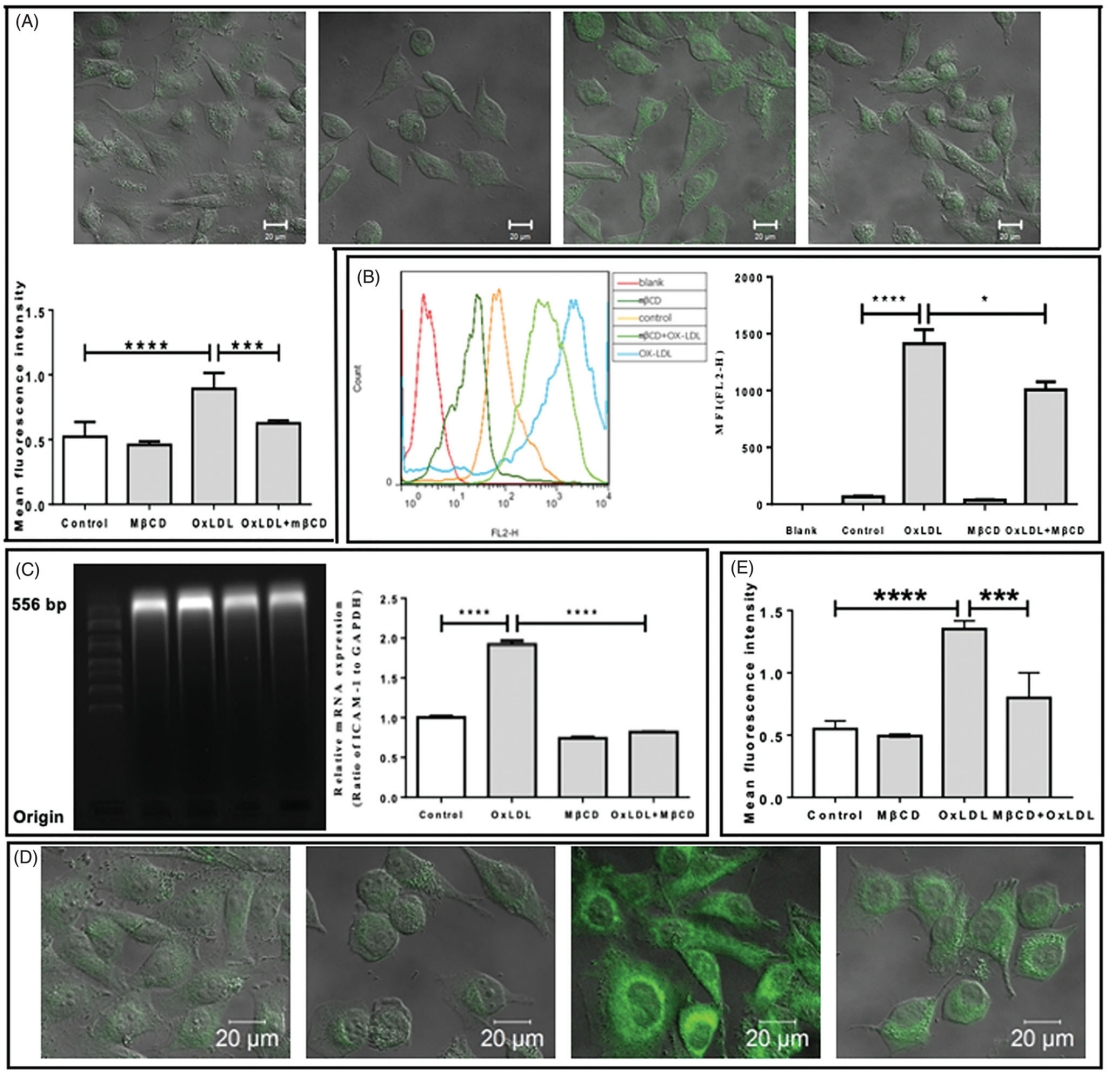
Inhibitory effect of MβCD on the oxLDL-induced expression of adhesion molecules in HUVECs. Four groups are categorized: HUVECs without treatment (control), treated with MβCD only (at 5 mM at 37 °C for 1 h), with oxLDL only (at 50 µg/mL at 37 °C for 12 h), and with both oxLDL and MβCD, respectively. (A) Confocal microscopic imaging of ICAM-1 molecules on cell surfaces (upper panel: representative confocal images; lower panel: quantification of MFI). (B) Flow cytometric detection of ICAM-1 molecules in cells (left panel: representative flow cytometric data; right panel: quantification of MFI). (C) The detection of ICAM-1 mRNA expression in cells. Left panel: agarose gel electrophoretic image; right panel: quantification of relative mRNA expression (ratio of ICAM-1 to GAPDH). (D) Representative confocal microscopic images of fluorescently stained VCAM-1 molecules on cell surfaces. From left to right: control, MβCD only, oxLDL only, and oxLDL + MβCD, respectively. (E) Quantification of VCAM-1 MFI. *p < 0.05; ***p < 0.001; ****p < 0.0001 compared with the corresponding group (*n* = 5 in A; *n* = 3 in B,C,E).

Then, the effect of MβCD on the LPS- (Figure 3) or oxLDL-enhanced (Figure 4) expression of ICAM-1 was investigated at both protein (Figures 3(A,B), 4(A,B)) and mRNA levels (Figures 3(C), 4(C)). In consistent with the abovementioned result, LPS alone or oxLDL alone induced a statistically significant increase in both protein level (a 2-fold increase in Figures 3(A), 4(A)) and mRNA level (a 3-fold increase in Figure 3(C) and a 2-fold increase in Figure 4(C)) of ICAM-1 on/in HUVECs. Interestingly, MβCD treatment reversed the LPS-/oxLDL-induced effect and significantly lowered the LPS-/oxLDL-stimulated ICAM-1 expression down to a level similar to that of the control (i.e., no significant difference compared with the control). The effect of MβCD on the LPS- (Figure 3(D)) or oxLDL-enhanced (Figure 4(D)) expression of VCAM-1 also was evaluated at the protein level by fluorescence staining and confocal imaging. The quantitative data (Figures 3(E), 4(E)) show that both LPS and oxLDL also induced an ∼2-fold increase in VCAM-1 expression and that MβCD treatment could reverse the LPS-/oxLDL-induced effect and significantly lowered the LPS-/oxLDL-stimulated VCAM-1 expression down to the control level.

### MβCD inhibited the LPS-/oxLDL-induced effects on pP65, IKK, and p-Akt

**Figure 5.**
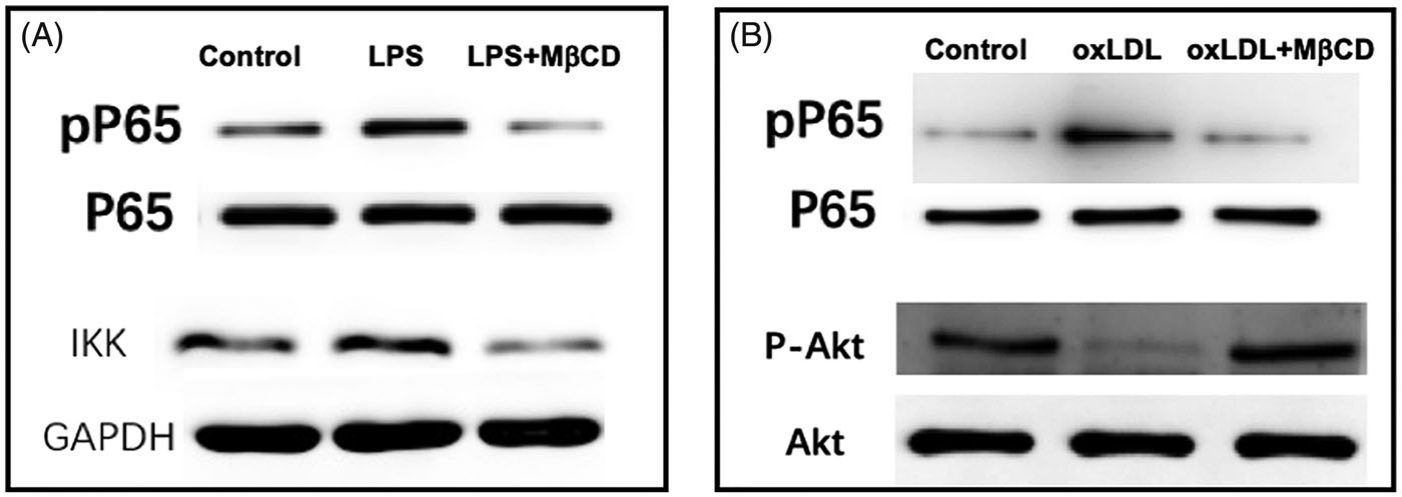
MβCD reverses the LPS- or oxLDL-induced changes in the contents of signal pathway key molecules in HUVECs detected by western blotting. (A) Western blots of P65 (a subunit of NF-κB), phosphorylated P65 (pP65), and IKK, respectively. Three groups are categorized: HUVECs without treatment (control), treated with LPS only (at 1 µg/mL at 37 °C for 12 h), and with both LPS and MβCD, respectively. (B) Western blots of P65, pP65, Akt, and phosphorylated Akt (p-AKT), respectively. Three groups are categorized: control, oxLDL only (at 50 µg/mL at 37 °C for 12 h), and oxLDL + MβCD, respectively.

Next, western blotting was performed to reveal the effects of MβCD on some key molecules in related signalling pathways. The data shows that both LPS and oxLDL induced the increase (∼2-fold) in phosphorylated nuclear factor-kappa B (phosphorylated NF-κB or phosphorylated P65 or pP65, Figures 5) and that LPS caused an increase (∼15% higher) in IκB kinase (IKK) (Figure 5(A)) whereas oxLDL impaired the phosphorylation of protein kinase B (phosphorylated Akt or p-Akt; ∼75% lower) (Figure 5(B)). Moreover, interestingly, it was also found that MβCD reversed the LPS- or oxLDL-induced changes in pP65 and IKK or p-Akt back to the control levels (Figure 5).

### MβCD suppressed the LPS-/oxLDL-induced effect on monocyte-endothelial adhesion

**Figure 6.**
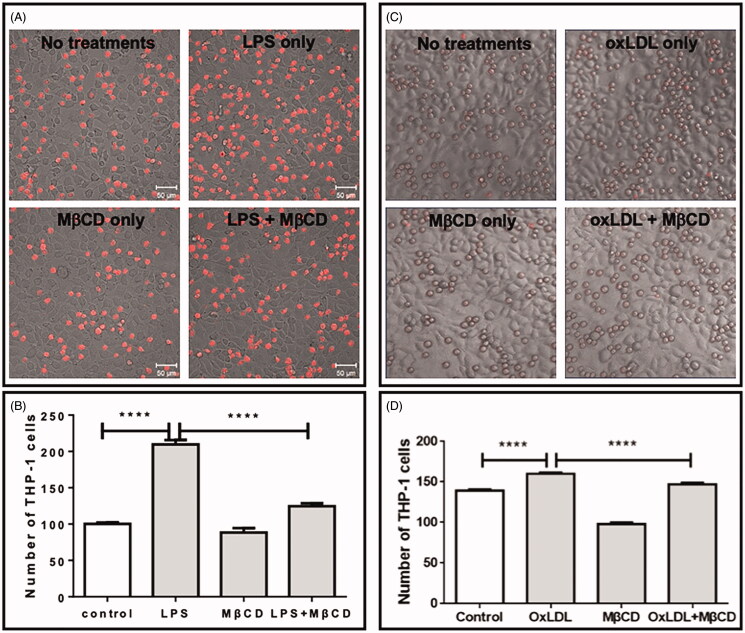
Inhibitory effect of MβCD on the monocyte-endothelial adhesion induced by LPS (A,B) or by oxLDL (C,D). The cells were treated without (control) or with LPS only (at 1 µg/mL at 37 °C for 12 h) or oxLDL only (at 50 µg/mL at 37 °C for 12 h), MβCD only (at 5 mM at 37 °C for 1 h), and LPS/oxLDL + MβCD, respectively. (A, C) Representative confocal images of fluorescently (CellTracker Red CMTPX) stained THP-1 monocytes on the monolayer of HUVECs. (B,D) Average numbers of THP-1 cells adhered on the endothelial cell monolayer in a field with the same size (****p < 0.0001 compared with the corresponding group; *n* = 4).

Finally, we tested the effect of MβCD on LPS- or oxLDL-induced monocyte-endothelial adhesion. The fluorescently stained monocytes (THP-1 cells) were incubated with the LPS-/oxLDL-activated endothelial cell monolayer pre-treated with or without 5 mM MβCD. Compared with the control without LPS/oxLDL stimulation, both LPS and oxLDL significantly improved the number of monocytes adhered on the endothelial cell monolayer (∼2-fold higher for LPS and ∼15% higher for oxLDL) whereas MβCD significantly suppressed the LPS-/oxLDL-induced change down to the control level (Figure 6).

## Discussion

Methyl-β-cyclodextrin (MβCD) has been mainly applied as a drug carrier in the pharmaceutical field (Loftsson and Duchene 2007) and as a cholesterol-depleting reagent in cell biological studies on lipid rafts (Ashrafzadeh and Parmryd 2015). It has also been reported that MβCD has potential antitumor efficacy and has the possibility of recruiting as an antitumor drug (Grosse et al. 1998a; 1998b; Gotoh et al. 2014; Resni et al. 2015; Elamin et al. 2018). Recently, accumulating evidence supports that cyclodextrins also have an anti-atherosclerotic efficacy (Zimmer et al. 2016; Ao et al. 2016a; Pilely et al. 2019; Wang et al. 2019; Kim et al. 2020).

Atherosclerosis is a chronic inflammatory disease and is closely correlated with inflammation caused by various reasons including bacterial infection or lipopolysaccharide (LPS; an endotoxin derived from the Gram-negative bacteria outer membrane) stimulation. During the initiation or development of atherosclerosis, the uptake of oxidized low-density lipoprotein (oxLDL) by multiple cells (e.g., endothelial cells, macrophages, and vascular smooth muscle cells) in the arterial tunica intima may induce lipid deposition in the cells or the formation of foam cells. Therefore, LPS- or oxLDL-stimulated cells are generally utilized as an *in vitro* cell model of atherosclerosis. However, it is unclear whether cyclodextrins exert the anti-atherosclerotic efficacy via influencing the LPS-/oxLDL-triggered monocyte-endothelial adhesion which is an important initial step of atherogenesis. By using MβCD as an example of cyclodextrins, this study sought to answer this question.

It is well-known that both LPS and oxLDL can promote the monocyte-endothelial cell adhesion via enhancing the NF-κB-mediated expression of multiple adhesion molecules including ICAM-1 and VCAM-1 (Myers et al. 1992; Cominacini et al. 1997a, 1997b; Takei et al. 2001; Li et al. 2014; Zhao et al. 2016; Deng et al. 2018). NF-κB (P65 is one of its subunits) is a transcription factor in an inactive state in the cytoplasm and the phosphorylation (activation) of NF-κB can result in the propagation of the response to the expression of ICAM-1/VCAM-1 in the nucleoplasm. IκB kinase (IKK) has been reported to be involved in the NF-κB-mediated expression of ICAM-1/VCAM-1 triggered by LPS (Sanlioglu et al. 2001). IKK can phosphorylate IκBα (Inhibitor of kappa B that keeps NF-κB in an inactive state in the cytoplasm via association with NF-κB) to release NF-κB for activation. Protein kinase B (Akt) has also been reported to be involved in the NF-κB-mediated expression of ICAM-1/VCAM-1 triggered by oxLDL (Ou et al. 2010; Huang et al. 2015). The dephosphorylation of Akt is also able to promote the activation of NF-κB via deactivating endothelial nitric oxide synthase (eNOS) which inhibits nitric oxide (NO) production (Ou et al. 2010).

Here, our data confirm that LPS could induce the upregulation of ICAM-1/VCAM-1 expression, NF-κB phosphorylation, IKK content, and monocyte-endothelial adhesion and that oxLDL could induce the upregulation of ICAM-1/VCAM-1 expression, NF-κB phosphorylation, and monocyte-endothelial adhesion, as well as the downregulation of Akt phosphorylation. Most importantly, we found that MβCD could reverse all of the LPS-/ox-LDL-induced changes. The results imply that MβCD can suppress the LPS- and oxLDL-triggered monocyte-endothelial adhesion via the LPS-IKK-NF-κB and oxLDL-AKT-NF-κB signalling pathways, respectively.

Presently, it is unknown why MβCD could influence NF-κB via different signalling pathways. MβCD is a cholesterol-depleting reagent and can disrupt lipid/membrane raft which is a sphingolipid-/cholesterol-rich microdomain mainly in the plasma membrane of cells and has been regarded as a platform for downstream signalling pathways (Chen et al. 2009; Levental et al. 2020; Ouweneel et al. 2020). A reasonable explanation is that MβCD regulates the signalling pathways by influencing the relationship between lipid raft and the receptors for external stimuli, e.g., toll-like receptor 4 (TLR4) for LPS and scavenger receptors (e.g., LOX-1 on endothelial cells or CD36 on macrophages) for oxLDL. In fact, it has been reported that the translocation of TLR4 into lipid raft is closely correlated with LPS-mediated inflammatory responses (Triantafilou et al. 2007; Villacorta et al. 2013; Fu et al. 2015) and that the uptake of oxLDL requires the recruitment of CD36 into lipid raft (Zeng et al. 2003; Rios et al. 2013). To clarify this speculation, further in-depth studies will be needed in the future.

Taken together, this study found that MβCD could impair the expression of adhesion molecules probably via LPS-IKK-NF-κB or oxLDL-Akt-NF-κB signalling pathway and then inhibit the LPS/oxLDL-enhanced monocyte-endothelial adhesion. Considering that the enhancement of monocyte-endothelial adhesion by a stimulus (e.g., LPS or oxLDL) plays a critical role in the initiation or development of atherosclerosis, our findings imply that cyclodextrin has the potential to inhibit atherogenesis by suppressing the LPS-/oxLDL-triggered monocyte-endothelial adhesion. MβCD is a widely used representative of cyclodextrins in cellular experiments and has structures and properties similar to other cyclodextrins, particularly hydroxypropyl-β-cyclodextrin (HPβCD) which is generally utilized in animal experiments. Therefore, the confirmation of the inhibitory effect of different cyclodextrins (e.g., MβCD and/or HPβCD) on LPS-/oxLDL-triggered monocyte-endothelial adhesion at the tissue or animal level can be performed in the future.

## Conclusions

In this study, the effects of MβCD on the LPS- or oxLDL-induced changes in expression/phosphorylation of adhesion molecules (e.g., ICAM-1 and VCAM-1) and related signalling pathway molecules (e.g., NF-κB, IKK, and Akt) and in monocyte-endothelial cell adhesion were investigated. It was found that MβCD significantly suppressed the LPS/oxLDL-triggered monocyte-endothelial adhesion by inhibiting the expression of adhesion molecules probably via LPS-IKK-NF-κB or oxLDL-Akt-NF-κB signalling pathway. The findings imply that cyclodextrin (e.g., MβCD) is probably able to exert an anti-atherosclerotic efficacy by suppressing the LPS-/oxLDL-triggered monocyte-endothelial adhesion. This study demonstrates a potential mechanism of the anti-atherosclerotic efficacy of cyclodextrins from the angle of monocyte-endothelial adhesion.
